# Expectation versus Reality: The Impact of Utility on Emotional Outcomes after Returning Individualized Genetic Research Results in Pediatric Rare Disease Research, a Qualitative Interview Study

**DOI:** 10.1371/journal.pone.0153597

**Published:** 2016-04-15

**Authors:** Cara N. Cacioppo, Ariel E. Chandler, Meghan C. Towne, Alan H. Beggs, Ingrid A. Holm

**Affiliations:** Division of Genetics and Genomics, The Manton Center for Orphan Disease Research, Boston Children’s Hospital, Harvard Medical School, Boston, MA, United States of America; University of Pittsburgh, UNITED STATES

## Abstract

**Purpose:**

Much information on parental perspectives on the return of individual research results (IRR) in pediatric genomic research is based on hypothetical rather than actual IRR. Our aim was to understand how the expected utility to parents who received IRR on their child from a genetic research study compared to the actual utility of the IRR received.

**Methods:**

We conducted individual telephone interviews with parents who received IRR on their child through participation in the Manton Center for Orphan Disease Research Gene Discovery Core (GDC) at Boston Children’s Hospital (BCH).

**Results:**

Five themes emerged around the utility that parents expected and actually received from IRR: predictability, management, family planning, finding answers, and helping science and/or families. Parents expressing negative or mixed emotions after IRR return were those who did not receive the utility they expected from the IRR. Conversely, parents who expressed positive emotions were those who received as much or greater utility than expected.

**Conclusions:**

Discrepancies between expected and actual utility of IRR affect the experiences of parents and families enrolled in genetic research studies. An informed consent process that fosters realistic expectations between researchers and participants may help to minimize any negative impact on parents and families.

## Introduction

As genomic technology becomes widely used in research to uncover the genetic influences on health and disease, there is a growing recognition of the importance of returning clinically-relevant genetic results to participants. In the United States, the National Institutes of Health (NIH) sponsors several research networks studying the implications of returning genomic data to patients (i.e., the eMERGE network http://www.genome.gov/27540473 and the CSER consortium https://cser-consortium.org), including to families of newborn infants (i.e., the NSIGHT network, http://www.genome.gov/27558493), yet the perceptions of families in receipt of such information in a research setting are largely unknown. Individual research result (IRR) return in genomic studies is a widely discussed topic [1–3], and it is clear that the promise of returning IRRs serves as an incentive for people to enroll in genetic research studies [4,5] and may persuade people who wish to gain personal benefit who otherwise would not participate [6]. This is especially true in pediatrics when parents may be on a desperate search for answers regarding their child’s undiagnosed condition. Ethics, perceived risk, family dynamics, socio-demographic factors, and characteristics of the diseases (including severity and potential preventability) are all factors that motivate parents to enroll their children in research. These factors also guide how investigators return IRR, inside or outside of genetic research studies [7–10]. Studies of the return of IRRs in children and adolescents with cancer suggest that parents and children have a strong desire to receive research results [10–12]. However, the discrepancies between expectations and the reality of the impact of results returned, especially for parents who have high expectations that the results may provide answers to their child’s condition, are unknown.

To date, most information collected on attitudes about returning research results in pediatric populations to parents is based on surveys using hypothetical scenarios and not actual IRR [13–15]. Not only may individuals respond differently to actual results, but the hypothetical scenarios do not include the consenting discussion that is part of any study offering return of actual IRR and is critical in setting parent expectations. Some argue that there is a need to expand the consent process to ensure that participants understand risks and other issues related to the return of IRR, but data assessing its role in expectation setting are lacking [16].

In this study we examined the expectations of parents who enrolled their child in a genetic research study, and relate how those expectations compared to the actual utility of the received results. We interviewed parents of children diagnosed with a rare disease, who enrolled in the Manton Center for Orphan Disease Research Gene Discovery Core (GDC) at Boston Children’s Hospital (BCH), a study of the genetic contributions to rare “orphan” diseases.

## Materials and Methods

### Recruitment

English-speaking parents of children enrolled in the GDC who received IRR and agreed to be contacted for future research at the time of consent were eligible for enrollment. Eligible families were contacted by the GDC genetic counselor (MT) and both parents were invited to participate. Each subject received a consent form and a letter describing the study in the mail and a research assistant (CC) consented parents by telephone. All signed consent forms were returned to the hospital. All clinical investigations have been conducted according to the principles expressed in the Declaration of Helsinkiwas and were approved by the Boston Children’s Hospital Institutional Review Board (protocol number IRB-P00011485).

### Telephone interviews

The research assistant (CC) conducted one semi-structured telephone interview with each parent (n = 9) to explore parents’ IRR return experience. A qualitative interview guide was developed which focused on six domains: Demographic information, influencing factors, results return experience, resulting decisions and actions, emotional and psychological effects, and reflection. Parents were asked to rate areas of satisfaction and emotion by use of structured scales and respond to open-ended questions regarding the six domains. (Survey available upon request.)

The interviews were conducted from May-July 2014 and each interview ranged from 30 to 90 minutes (mean, 54 minutes; standard deviation, 20 minutes). The interviewer took notes and audio-recorded the interview for accurate data collection. Since the interviews were carried out by telephone, parents were asked to remain in a private location for the duration of the interview.

### Analysis

Structured data from the interviews were recorded and unstructured data were transcribed verbatim from the audio recordings. The transcripts were independently reviewed by two research assistants (CC and AC) to generate a list of common themes and emotions that surfaced. The lists were reconciled to create a catalog of the most prevalent themes and emotions, which was used to create a coding scheme. Many of the codes were assigned sub-codes to distinguish pre-IRR return, post-IRR return, or both. The two research assistants then independently coded each transcript using the coding scheme. Coded comments were compared *post hoc* and discrepancies were resolved by consensus. Codes were organized by parent within each category to ensure that overall theme frequencies were not overrepresented from one parent discussing the same theme many times. Data were analyzed using descriptive statistics and recurring patterns were identified. Relationships between the codes, within and across individual transcripts, were recorded.

## Results

### Participants

At the time of this project, there were 27 probands enrolled in the GDC for whom IRR had been returned to the family. The return of IRR ranged from 6 months to 2 years prior to the telephone interview. After screening for English-speaking families who stated in their consent that they were open to contact for additional studies, 7 two-parent families (14 parents) were contacted by the GDC genetic counselor. Of the 14 parents, 9 (64.3%) participated: 3 couples and 3 individual parents (6 families represented in total). The main reason parents cited for declining is that they were too busy and did not have the time. Table 1 lists the parents (1 through 9), diagnoses of the probands, and who was interviewed (mother or father). The number after each quote below refers to the parent as designated in Table 1. See Table 2 for demographic information. No statistically significant associations were identified between demographic variables and our findings.

**Table 1:** Parent numbers.

Parent Number	Mother or Father	Child’s diagnosis
1	Mother	Ohtahara syndrome
2	Father	Ohtahara syndrome
3	Mother	Floating-Harbor syndrome
4	Father	Floating-Harbor syndrome
5	Mother	Mutation in *OPHN1*
6	Mother	Floating-Harbor syndrome
7	Mother	Episodic Ataxia Type I
8	Father	Cantú syndrome
9	Mother	Cantú syndrome

**Table 2:** Participant and Child Demographics.

	Parent	Child
**Age**		
Mean	43.6	10.8
Range	31–58	5–20
**Gender**		
Male	3 (33.3%)	7 (77.8%)
Female	6 (66.7%)	2 (22.2%)
**Race**		
White	9 (100%)	9 (100%)
**Relationship of Parent to Child**		
Biological Father	3 (33.3%)	
Biological Mother	6 (66.7%)	
**Highest Level of Education**		
Trade School	1 (11.1%)	
Some College	1 (11.1%)	
2 or 4 Year College Degree	3 (33.3%)	
Post-College Degree	4 (44.4%)	
**Self-reported Knowledge of Genetics**		
Poor	2 (22.2%)	
Fair	6 (66.7%)	
Good	0 (0.0%)	
Excellent	1 (11.1%)	
**Prior Experience with a Genetic Research Study**		
Yes	2 (22.2%)	
No	7 (77.8%)	
**Number of Children in Family**		
1	2 (22.2%)	
2	4 (44.4%)	
3	2 (22.2%)	
4	0 (0.0%)	
5	1 (11.1%)	
**Family History of Genetic Diseases**		
Yes	7 (77.8%)	
No	2 (22.2%)	

IRRs were identified as follows: 1) whole genome sequencing of the proband only (1 family), 2) whole exome sequencing of the proband and 2 parents (3 families), and 3) targeted sequencing of a candidate gene (2 families). None of the probands had a positive family history for their condition, and all mutations were confirmed to be *de novo*.

### Utility of IRR

Each parent shared thoughts about the usefulness of receiving their child’s IRR and the ways in which their family planned to utilize, or has utilized, the information. Five themes emerged from the data:

Predictability–ability to predict the child’s future health, medical care, opportunities, life expectancy, etc.

Management–how the condition and symptoms are monitored and treated

Family planning–future reproductive planning of any family member, including the parents, affected child, or other children in the family

Finding answers–the family’s desire to receive any answers to remaining questions about the affected child’s medical health and/or diagnosis

Helping science and/or other families–benefit that scientific research or other families might gain as a result of the parent’s participation in the GDC.

#### Expected utility of IRR

The GDC consent form provides parents the option to choose whether they would like to receive their child’s IRR, and participants were asked what motivated them to choose to have IRR returned. Three parents said they expected the information to help them predict the future of their child or family. *“I’ve always believed that [child’s] life would be shortened as a result of his diagnosis*. *To have predictability was so important to me […] and knowing what his services and his needs might be in the future”* (5). Another parent shared a similar desire, stating that even if there was *“nothing to be done [with the results returned]*, *at least we have that knowledge and could prepare for what is happening”* (7).

Three parents hoped that the IRR returned would provide information that would help them manage their child’s health. *“Maybe it will help him with funding or when he gets older with disability*, *being able to get disability programs that he wasn’t able to before*….*I feel that that could be helping him later on*, *versus saying ‘we don’t know what he has*, *he has like 3000 issues*, *but we don’t know what he has’”* (9).

Three parents reported that they expected the results returned to provide information about family planning. *“We were still wanting to grow our family and just wanted to be prepared … we thought*, *‘OK*, *we don’t want to put any future suffering on any children”* (1). Another parent shared her concerns about the reproduction of her children, sharing that her and her husband *“wanted to know if his brother needed to be concerned about passing this onto his children”* (5).

All parents expressed a desire to find answers pertaining to their child’s condition in order to put a name to it. A mother remembers where her family was in their journey when they enrolled in the GDC study: *“I think we felt like we’ve sort of exhausted all the diagnostics we can to understand what’s happening*, *and that was sort of where we were when we ended up going into The Manton Center”* (7). One father explains that *“up until that point it was an unknown genetic syndrome*, *and we wanted to confirm the exact diagnosis*,*”* (4), and a mother shared the sentiment, *“we were just wondering the origin or the cause of it”* (6). Another mother simply exclaimed, *“I can’t imagine not knowing!”* (5).

For several parents the benefit for science and other families was a strong motivator for participating in the GDC. One mother explains, *“it’s nice to know something*. *But we also figure participating can further science and help kids in the future if not [child]*, *so that’s why we were kind of okay with however it turned out*,*”* (1). Some parents anticipated helping others who had similar results: *“We would like to get in contact with [other families] and help them out*. *Like a single mother or a single father”* (8).

#### Actual utility of IRR

As parents discussed the experience of receiving results, they described the actual utility they gained from learning their child’s. Two parents stated that the results provided some predictability about their child’s future. One father explains that *“the results were negative [emotionally]*, *[but] the positive was*, *‘okay so we have the ability to affect the future’”* (4), whereas a mother learned that her son’s condition *“is something that people do live with*. *It’s not some purely degenerative neurologic condition*. *On some level it is*, *but it’s not like*, *we weren’t getting an MD diagnosis like there was only one way you’re going”* (7).

Three parents reported that the IRR allowed them to plan the management of their child’s condition and medical care. One mother explains that after choosing to share the IRR with her child’s medical care team, *“the [results] reinforced making a change and taking him off the generic seizure medication and putting him on the name brand”* (5).

Only 3 parents expected the IRR to inform family planning, yet 5 parents reported that the IRR had an impact on their family planning decisions, all in the context that the mutations identified were all *de novo* with low risk for recurrence. *“First*, *we thought we might have to do some limiting factors*,*”* … *“But now I‘d say it makes us*, *if anything*, *want to have more kids because we want to have plenty of support around [child]”* (1). Some parents found information on family planning to be useful for the next generation: *“[The genetic counselor] brought a few articles and then she started showing me*, *and she said ‘well here’s a family tree*,*’ […] And I remember her saying*, *‘if [child] was old enough to have children*, *he’d have about a 50% chance of even passing it on’*. *And those were things we just never even thought about”* (7). Another parent shares, *“we wanted to know if his [affected child’s] brother needed to be concerned about passing this onto his children*. *[…] And when the doctor told my husband that this wasn’t something he had to be concerned about*, *my husband wept*. *It was relief*.*”* (5). Alternatively, irrespective of recurrence risk, for one family having prognostic information informed by the IRR made them feel that growing their family was no longer an option: *“We would not have any more children because it would not be fair to [child] or the other child*, *because [child] requires a lot of attention”* (8).

All parents said that receiving the IRR provided them with answers about their child’s medical condition. For some, receiving a genetic answer was met positively; as one mother explained, *“There’s a little reassurance*, *relief*. *At least we can put a name to it*, *that we’re not the only ones out there*.*”* (8). Some parents were both surprised and comforted by the genetic answers, such as one mom who was *“pretty blown away”* and explained, *“it was pretty amazing because we could then say*, *‘oh my God*, *this does explain [child] and why he learns the way he does*, *and why he can’t do X*, *Y and Z*. *This explains so much about him*.*’”* (5).

One father expressed mixed emotions about whether or not there would be actual utility from receiving an IRR: *“It’s just given us some reassurance [that] it’s something and not a complete unknown*. *But I guess because the future’s still unknown*, *even though we have a name to it*, *I guess that’s sort of a problem too*.*”* He continues to explain: *“[…] it really didn’t change anything at all other than giving a name to what we were dealing with”* (4). This father’s disappointment with the lack of utility of the results, despite finding answers, was shared by his wife who explained, *“when I did get the confirmation that [child] did have Floating Harbor*, *I guess like a chapter in that book had closed but I still don’t know what lies ahead for her future”* (3). While all parents expressed gratitude for having an answer and a diagnosis, several of them shared the feeling that it was *“just an answer*, *nothing else changed”* (8).

One mother was able to directly help other families with the IRR returned through the GDC. Using the IRR and information learned following the results, she was able to share the benefits with an online support group that she had created prior to participation. *“It was really nice to finally have some answers and finally have a test that when people came to us with questions*, *we could say ‘here’s a test and you can find out for sure without waiting years and wondering what would happen’*, *so that was real positive”* (6).

Some parents expressed their understanding of the limitations they face when participating in rare disease research. *“This might explain something and give some kind of closure or peace of mind*, *which is good*, *but there’s no like*, *‘OK*, *so because of this gene mutation*, *here’s a pill*, *or here’s a treatment*, *or we can do gene therapy or…’ you know there’s always that hope maybe they’ll come up with something*. *But there’s not like a direct*, *immediate response*. *There’s no treatment”* (1). Some even acknowledged that their expectations for the utility of the study might not be realistic. *“I guess maybe I didn’t realize just how extremely rare [child’s] diagnosis was going to be*. *And I maybe really set the bar too high in my expectations and my assumption*, *that I was going to be able to connect with other families”* (5).

#### Actual utility of IRR as reported by the Manton Center genetic counselor

Parents 1 and 2 are the parents of a now seven-year-old child who was originally enrolled at 20 months with unexplained seizures. His initial work up suggested a possible mitochondrial component to his disorder, which was later determined to be a secondary finding, possibly resulting from a medication. The IRR the family received lead to an understanding that his condition is primarily neurological, and that further metabolic work up was not indicated.

Parents 3, 4 and 6 are the parents of individuals who were enrolled with a clinical diagnosis of Floating Harbor syndrome (FHS), and for whom targeted sequencing of the newly identified *SRCAP* gene confirmed this diagnosis molecularly. These families were part of a larger cohort of 16 individuals sent from our Center with a clinical diagnosis of FHS. The results of this were published in a multi-centered, collaborative paper, which has been cited in several papers about the phenotype of FHS.

Parent number 5 is the mother of a 20-year-old with a mutation in *OPHN1*, resulting in an X-linked mental retardation syndrome. As expressed in her responses, one of the primary utilities she hoped to gain was to be connected with other families. At the time that this interview was performed, she had not successfully connected with any other families. However, through the efforts of research staff and family advocates, there are now at least six families from around the world with *OPHN1* variants who are connected.

The child of parent number 7 is a nine-year-old boy who was diagnosed with Episodic Ataxia Type 1 (EA1). The return of the IRR lead to treatment with an indicated seizure medication which was found to have variable effects on ameliorating features in individuals with EA1. While this did reduce the severity and the frequency of his episodes, he experienced severe behavioral issues while on the medication. These side effects were so disruptive that the medication was discontinued. This was a difficult experience for the family, as what originally seemed like a benefit to participation, ended up not being a benefit.

Lastly, parents 8 and 9 are parents of an eight-year-old boy who was enrolled with a clinical diagnosis of Zimmermann-Laband syndrome; however exome sequencing revealed a molecular diagnosis of Cantú syndrome. Due to the phenotypic overlap between the conditions and the extensive clinical work-up, which the participant had already received, no additional referrals or assessments were indicated. The study staff attempted to connect the family with other families at an in-person event, and the publishing of the case report resulted in the diagnosis of another individual.

Of the five themes which emerged from the participants’ interviews [i.e. I) Predictability, II) Management, III) Family planning, IV) Finding answers and V) Helping science], both “Family planning” and “Helping science” were universally achieved in this cohort. The IRR allowed recurrence risk counseling to be provided. Further, because all the findings in this group were *de novo*, no additional familial testing was indicated. All of the families helped science as they have been included in manuscripts submitted for publication, thus advancing scientific knowledge on these rare conditions and rare genetic variants. Resulting publications are shared with families after publication.

### Emotions and Satisfaction as Related to Expected and Actual Utility of IRR

#### Categorizing parent emotions

Parents were categorized as “Positive Emotion Parents,” “Negative Emotion Parents,” and “Mixed Emotion Parents” (Positive and Negative) based on the frequency of positive and negative “post-IRR return,” relative to “pre-IRR return,” emotional codes during their accounts of the IRR return experience within their transcripts. Five parents (3 families, 2 couples) were classified as “Negative Emotion Parents” and expressed a great deal of uncertainty about their child’s diagnosis and future, as well as disappointment with the availability of medical information. Negative emotion codes included frustration, hopelessness, anxiety, depression, stress, and disappointment. Three parents (2 families, 1 couple) were classified as “Positive Emotion” and predominantly discussed having strong support systems, open communication with doctors, and control over the management of symptoms for their children. Frequently coded emotions for these parents included gratefulness, relief, hopefulness, and empowerment. One parent was classified as “Mixed Emotion” and expressed positive as well as negative emotions.

#### Parent emotion and utility of IRR

We compared the parental views of expected and actual utility between emotion groups. We found a consistent association between emotion group and reported utility among all parents (Table 3). “Negative Emotion” and “Mixed Emotion” parents reported that the utility of the IRR they received was either less, or different, than expected. On the other hand, “Positive Emotion” parents reported that the utility of the IRR matched, or even exceeded, their expectations.

**Table 3:** Parent Emotion Categories and Actual vs. Expected Utility Groupings.

	Actual Utility Less Than or Deviates From Expected Utility	Actual Utility Greater Than Expected Utility
**Positive Emotion**	0	3
**Mixed Emotion**	1	0
**Negative Emotion**	5	0

#### Parent satisfaction and utility of IRR

We asked parents to rate their satisfaction with 1) the process of return of results, 2) the amount of genetic information returned, 3) the level of detail of the genetic information, and 4) the resources available after IRR return. Most parents (6 of the 9) reported that they were “Satisfied” or “Very Satisfied” on all of the questions. The remaining 3 parents reported feeling “Dissatisfied” or “Very Dissatisfied” on at least one of the four satisfaction questions described above, and were the same 3 parents who fell into the “Negative Emotion” category. To assess regret regarding the overall IRR experience, we asked parents, “If you could re-do the Manton research study experience, would you choose to have your child’s results returned again?” All 9 parents said they would participate in the process and receive IRR again.

## Discussion

A number of studies exploring the hypothetical return of IRR have shown that parents’ desire to receive research results is a motivator for participation in pediatric genetic studies [13–15]. This motivator was unanimously reported as perceived utility in the current study. Also similar to our findings, prior studies cite “facilitating financial and emotional planning” and “informing future decisions and medical care” as areas of perceived utility of IRR [15]. However, many individuals in these studies also expressed deep discomfort with the idea of not learning information from the results and had concerns about financial burden and discrimination [14,15]. Some of these logistical and emotional concerns were realized for parents in the current study, as their personal experiences shed light on the direct positive and negative impact of IRR. To our knowledge, the current study is one of the first to explore parental perspectives on the actual return of IRR through rare disease research. While a desire to receive IRR was consistent across parents interviewed, the discrepancy between expected and actual utility varied among the parents.

All nine parents received utility from finding answers to questions, mainly to put a name to their child’s disease. Over half of the parents found the results useful in their family planning; all results returned were *de novo* mutations and therefore, none of the families had a significant recurrence risk. In terms of predictability and management, some parents found that the IRR provided a clearer view of the progression of their child’s health and allowed them to make more informed decisions for monitoring and treating disease symptoms. Many parents elected to share the results with their family, friends, and other healthcare professionals. One mother reported that sharing results online helped other families find a diagnosis and encouraged participation in rare disease research studies.

The three themes: predictability, management and finding answers, were important to all families with rare disorders but are not always achievable. Practice guidelines and treatment standards are often not available due to the scarcity of cases and lack of comprehensive studies. Families described in this paper were all enrolled in a gene discovery research project, and either had ultra-rare conditions (Cantú syndrome, *OPHN1*), conditions where the genetic cause is newly described (Floating Harbor syndrome), or non-classical presentations of known conditions (Episodic Ataxia type 1 and Ohtahara syndrome). Families with rare, undiagnosed conditions who receive IRR are faced with learning this information as a result of cutting edge research, suggesting that these will be the exceptional cases, not diagnosed through traditional clinical testing. For many such IRRs, there may be even less known about a result, making prognosis and management more difficult. While developing practice guidelines will take time and requires a comprehensive review of several individuals with a given rare condition, the area in which medical professionals can provide assistance to families more efficiently is by “Finding answers” through by connecting families with other families with the same diagnosis.

Consistently we found that the parents classified in the “Negative Emotion” group were those who expected more utility from the IRR than they felt they actually gained. The parent classified in the “Mixed Emotion” group gained the same utility as expected, but in different areas than expected, (i.e. IRR was expected to aid in their child’s management, but instead the results helped in predictability). Conversely, “Positive Emotion” parents’ actual utility was as much, or greater, than they expected. The fact that 6 of the 9 parents (4 of 6 families) fell into the “Negative Emotion/Mixed Emotion” group was concerning.

One of the main differences between the “Positive Emotion” and “Negative Emotion/Mixed Emotion” groups was related to prior expectations in specific areas of utility. None of the parents in the “Positive Emotion” group expected the results to provide predictability and guide management, whereas half of the “Negative Emotion/Mixed Emotion” parents expected the results to do that. Of the "Negative Emotion/Mixed Emotion" parents, only one reported being able to use the IRR to aid in her child’s health management and one parent reported that the IRR helped with predictability of the child’s disease. The largest discrepancy between expected and actual utility of the IRR for the “Negative Emotion” parents was primarily due to the expectation that the results would help predict disease progression and reveal the best management plan for their child, which was generally not fulfilled.

These findings can also be applied to recent attention toward personalized medicine. In the pediatric clinical setting, genetic research results have been shown to have considerable impact on children’s health outcomes, finding effective interventions, and finding patient support services for families [16]. However, the return of IRR to research participants has been debated due to legal and ethical differences between research and medical care and concern about triggering therapeutic misconceptions [17]. Further research regarding the latter point will be useful as genetic research results overlap into the clinical setting. All 9 parents in our study were willing to re-do the experience and none reported regret about receiving IRR, revealing the high value placed on individualized genetic results from the parental perspective, as well. However, there exists a lack of understanding about what those results will accomplish, with only 3 parents having an overall positive emotional experience with the IRR return mostly due to a disconnect between expectations and actual utility received. Although all parents were informed during consent that results may not be found and may not provide any additional information, utility expectations varied. Adopting a “one-size fits all” approach for handing genetic results should be avoided due to the differing perceptions of IRR amongst parents [15]. Informed consent in research is instrumental in shaping the expectations of both the study participants and research personnel [18,19], but the concept of “personalized” or “individualized” results may cause subjects to misconstrue the amount of benefit it will bring them. A section during consent specifically outlining realistic expectations in more detail could encourage participants to take the time to consider their own personal beliefs and expectations, and to better understand what the study realistically can offer in terms of IRR utility. If the study has returned IRR to participants in the past, researchers might also find it useful to provide evidence of real-life experiences by other families who have received IRR. Practicing this type of structured communication within the informed consent process may help parents and families generate reasonable expectations for IRR utility. Further research should be done to examine how the personalization of the return of results experience makes individuals more likely to inflate the benefits, and ways that this can be combated.

It is also important to acknowledge how resources and support available to families following IRR return can help maximize the utility received. Support groups and contact with other families in similar situations may aid in predicting the progression of a child’s disease or learning options for management. Finding support for families with rare disorders can be a challenge due to the small number of families diagnosed with the disease and to geographic limitations. While all parents indicated that they had access to various sources of support, 6 of the 9 parents interviewed were unsatisfied and disappointed that the results did not make it easier to find a diagnosis-specific support group. To combat this issue, four parents suggested creating a central database or resource online, where families can go to find others for support, advice and guidance in regard to their child’s genetic disease. The GDC has plans in place to develop an interface to connect families with rare diseases to provide a greater network for support.

There are limitations to our study. The sample was a small, select group of parents who have children with rare genetic diseases. Their unique experience with the long road to diagnosis might account for their frustration, uncertainty and desire to find as many answers as possible regarding their child’s medical condition. Future studies may benefit from measuring baseline emotional status of the participants before receiving IRR in order to better understand how preexisting emotions may influence emotional status after receiving IRR. Recall bias may have also affected parent reports of the IRR return experience, since all results were returned 6 months to 2 years prior to conducting the telephone interviews. In addition, all parents interviewed were Caucasian and married, resulting in a less representative sample. Future studies involving a larger sample and greater variety of genetic diseases are needed to assess if the expected and actual utility, and the emotions that accompanied them, are generalizable to a greater population.

Our study provides insight into the expectations and experiences of IRR utility for families enrolled in rare genomic disease research, and highlights the need for an informed consent process that fosters realistic expectations and effective communication between researchers and participants. Attention to these issues will become increasingly important as researchers begin to disclose results from an increasing array of pediatric genomic research projects.

## Supporting Information

S1 Table Table of complete code counts, by subject. Table of codes found in interviews, for each parent interviewed and sorted further by timeframe (pre or post return of result/ or neither).
(XLSX)

S1 Text Interview Coding Schema. List of Codes found in interviews, sorted by theme.
(DOCX)
