# Supplementary material for: Expectation versus Reality: The Impact of Utility on Emotional Outcomes after Returning Individualized Genetic Research Results in Pediatric Rare Disease Research, a Qualitative Interview Study
Source: PLoS One. 2016 Apr 15;11(4):e0153597. doi: 10.1371/journal.pone.0153597 (PMC4833284; doi:10.1371/journal.pone.0153597)
Supplement: S1 Text — List of Codes found in interviews, sorted by theme. (DOCX) [file pone.0153597.s002.docx]

**Parent Interview Coding**

**A. Parent**

1. Role in Child’s Medical Care
   1. Proactive/Advocate
   2. Information seeker
   3. Passive
2. Attitude toward Medical Professionals
   1. Confidence
      1. At BCH
      2. In general
   2. Skepticism
      1. At BCH
      2. In general
   3. Reliance
      1. At BCH
      2. In general
   4. Distrust
      1. At BCH
      2. In general

‘ Pre results return/ ‘’ Post results return

**B. Communication**

1. Form of Communication
   1. Phone
   2. Email
   3. Face-to-Face
2. With Whom
   1. Doctor(s)
   2. Genetic Counselor
   3. Research Team
   4. Family/Friends
3. Regarding
   1. Genetic results return
   2. Child’s medical health in general
   3. Follow-Up about the study
   4. Further genetic testing
   5. Options Available
4. Information Sharing (about genetic results)
5. With doctors, genetic counselors, medical professionals
6. With family/friends
7. With school
8. With other researchers

‘ Pre results return/ ‘’ Post results return

**C. Family Dynamic**

1. Communication
2. Siblings
3. Spouse
4. Extended family

‘ Pre results return/ ‘’ Post results return

**D. Utility**

1. Personal Utility
   1. Predictability
   2. Management
   3. Family planning
   4. Find answers/closure

‘ Expected/ ‘’ Actual

**E. Support/Resources**

1. Medical Information
2. Personal Access to Medical Information
   - 1. Journal articles
     2. Web pages
     3. Other parents/families/friends
3. Medical Information provided by Medical Professionals
   - 1. Journal articles
     2. General education
4. Referrals to Other Medical Professionals
5. Organizations (providing financial support/items families could not otherwise obtain – wheelchairs, bikes, equipment)
6. Availability/Time of parent
7. Emotional Support (indicate “Available” or “Desired”)
   1. Family/Friends
   2. Mental Health Treatment
      1. Therapist/Counselor/Social Worker
      2. Medication
   3. Religion/Faith
   4. Find Answers/Have closure
   5. Support Group
   6. Big support group
   7. Small support group
   8. Online
   9. In-person
   10. Specific to diagnosis
   11. Not specific to diagnosis

‘ Available/ ‘’ Desired

**F. Action due to results return**

1. Change to child’s medical treatment (management, medications, etc.)
2. Change to logistical planning (insurance plans, finances, etc.)
3. Finding Support for Self (seeking out support group, therapist, family/friends, etc.)
4. Providing Support for Others (running a group, blog, etc.)
5. Further Genetic Testing
   1. On self
   2. On child
   3. On Immediate Family
   4. On Extended Family

**G. Uncertainty**

1. About the diagnosis
2. About the management
3. About the genetic results returned
4. About the prognosis/future

‘ Pre results return/ ‘’ Post results return

**H. Understanding (of the Manton Study)**

1. Consent form
2. Study procedures
3. Meaning of results returned
4. Limitations of the study/genetic testing
5. Time frame of the study

‘ Pre results return/ ‘’ Post results return

**I. Emotion**

1. Positive
   1. Joy
   2. Relief
   3. Grateful
   4. Hopeful
   5. Comfort
2. Negative
   1. Disappointment
   2. Fear
   3. Frustration
   4. Hopelessness
   5. Anger
   6. Anxiety
   7. Depression
   8. Guilt/Blame
   9. Overwhelmed
   10. Devastated
   11. Difficult/Hard
3. Mixed (Positive & Negative)
4. Neutral
5. Numb

‘ Pre results return/ ‘’ Post results return
